# Evaluation of the Diagnosis and Antibiotic Therapy of Sepsis in the Emergency Department: A Retrospective Observational Study

**DOI:** 10.3390/biomedicines13071566

**Published:** 2025-06-26

**Authors:** Eszter Varga, Sándor Somodi, Máté Molnár, Dóra Ujvárosy, Krisztina Gaál, Attila Vaskó, Zoltán Szabó, Ildikó Bácskay, István Lekli, Adina Fésüs

**Affiliations:** 1 Doctoral School of Pharmaceutical Sciences, University of Debrecen, H-4032 Debrecen, Hungary; varga.eszter@pharm.unideb.hu; 2 Department of Pharmacodynamics, Faculty of Pharmacy, University of Debrecen, H-4032 Debrecen, Hungary; molnar.mate@mailbox.unideb.hu (M.M.); lekli.istvan@pharm.unideb.hu (I.L.); 3 Department of Emergency Care and Oxyology, University of Debrecen, H-4032 Debrecen, Hungary; somodi@med.unideb.hu (S.S.); ujvarosy.dora@med.unideb.hu (D.U.); szabo.zoltan@med.unideb.hu (Z.S.); 4 Department of Internal Medicine, Faculty of Medicine, University of Debrecen, H-4032 Debrecen, Hungary; gaal.krisztina@med.unideb.hu; 5 Department of Pulmonology, Faculty of Medicine, University of Debrecen, H-4032 Debrecen, Hungary; vasko.attila@med.unideb.hu; 6 Department of Pharmaceutical Technology, Faculty of Pharmacy, University of Debrecen, H-4032 Debrecen, Hungary; bacskay.ildiko@pharm.unideb.hu

**Keywords:** albumin level, antibiotic, guideline adherent, length of stay, risk factors, sepsis, survival, 30-day mortality

## Abstract

**Background/Objectives**: Sepsis is one of the most common causes of death worldwide, and its diagnosis remains a challenge for clinicians. The main purpose of this study was to appraise the diagnosis and antibiotic prescription pattern for sepsis admitted to the Emergency Department (ED), comparing Sepsis-2 to Sepsis-3 criteria. **Methods**: The study was conducted in an ED of a tertiary care medical center in Hungary. We included all adult patients who were diagnosed with sepsis in 2023. Data collection was made manually from UD MED System. Diagnosis was assessed based on Sepsis-2 and Sepsis-3 criteria, then compared. Further analyses were made only in cases with confirmed sepsis diagnosis. Antibiotic guideline adherence was determined according to the local guideline in force. Fisher’s exact test, *t*-test, and ANOVA were applied to compare categorical and continuous variables between groups. The Kaplan–Meier test was applied for probability of survival. Significant *p*-values were defined as below 0.05. **Results**: The substantial majority of patients recorded with sepsis in the ED met both the Sepsis-2 and Sepsis-3 criteria (80%), while the rate of misdiagnosis was similar (Sepsis-2: 16/91, 17.6% and Sepsis-3: 14/91, 15.4%). The most important identified risk factors in sepsis were old age (60+ years) and comorbidities (CCI ≥ 4). Elevated LDH (median 325 mg/dL) and decreased albumin levels (median 26 g/L) can be used as early indicators of sepsis. Although the time to first antibiotic administration was not associated with significantly better clinical outcomes, the guideline-adherent agent selection (Sepsis-2: 18/43, 41.9% and Sepsis-3: 19/46: 41.3%) led to a significantly longer survival (median 37 vs. 4 days). **Conclusions**: No significant differences were found in diagnostic accuracy or prediction of mortality between Sepsis-2 and Sepsis-3. Guideline-adherent antibiotics may lead to significantly higher survival rate in sepsis.

## 1. Introduction

Sepsis is one of the leading causes of death worldwide. In 2017, 11 million deaths were reported from the 48.9 million sepsis cases, accounting for 19.7% of all deaths worldwide [1]. Moreover, sepsis represents the first cause of death for infection, estimating that 15 out of every 1000 hospitalized patients will develop sepsis as a complication of receiving healthcare, accounting for 17% of in-hospital mortality [2,3].

According to the 2024 summary report of the European Center for Disease Prevention and Control (ECDC), in 2021 out of the 73,872 patients who spent more than two days in an intensive care unit (ICU), 11,551 (15.6%) developed a hospital-acquired infection (HAI) of which 7.1% (5707) cases were sepsis. However, in Hungary, this rate was almost double (13.6%) [4]. In the USA, 86% of sepsis is diagnosed on admission, out of which 80% receive the first treatment in the Emergency Department (ED) [5]. In recent years, significant progress has been made in the treatment of sepsis, but sepsis remains with the highest mortality rate in the ED, which is primarily due to diagnostic difficulties and its rapid progression [6].

According to the Sepsis-3 (Third International Consensus Conference 2016) definition, sepsis is a “life-threatening organ dysfunction caused by a dysregulated host response to infection” [7], a condition that significantly complicates its early diagnosis. However, early diagnosis and the consequent appropriate treatment significantly reduce sepsis mortality [8]. Therefore, diagnostic criteria have been introduced that help to establish a rapid diagnosis based on the patient’s current condition and parameters. At the same time, experts are divided on which diagnostic criteria may lead to better clinical outcomes. Although the predictive validity of Sequential Organ Failure Assessment (SOFA) for in-hospital mortality in the ICU was statistically greater than Systemic Inflammatory Response Syndrome (SIRS)—Sepsis-2 (International Sepsis Definitions Conference, 2001) criteria—and Quick Sequential Organ Failure Assessment (qSOFA) criteria, outside the ICU, the qSOFA score was statistically stronger than SOFA and SIRS criteria, supporting its use as a prompt to consider possible sepsis [9]. Spoto et al. found that outside the ICU, the best diagnostic tool for sepsis is combining SIRS criteria or qSOFA score with procalcitonin (PCT) and Mid-Regional pro-Adrenomedullin (MRproADM) serum levels [8]. Another retrospective cohort study among adult patients with suspicion of sepsis at the ED found an association between higher qSOFA scores and ICU admission [10].

Furthermore, attempts to replace SIRS criteria with a qSOFA score for the assessment of potential septic patients are problematic. The qSOFA score should be considered as a prognostic rather than a diagnostic tool. Some experts argue that the qSOFA score does not meet the definition of a diagnostic assessment tool, and thus, SIRS assessment remains the gold standard for diagnosis in the ED [11].

Nevertheless, scoring systems that require a limited number of clinical parameters, such as a qSOFA score, combined with biomarkers (e.g., PCT) that are already routinely available in the ED, are preferred [12]. Considering that the average hospital cost of sepsis treatment was $32,000 per patient in 2016, rapid and accurate diagnosis is of utmost importance, as it may result in significantly decreased length of stay (LOS), thus reducing the financial burden on the healthcare system [13].

Several studies demonstrate that early diagnosis followed by appropriate antimicrobial treatment is essential in sepsis, especially in septic shock, as even very short delays can greatly influence clinical outcome [9,14]. A large retrospective study including 35,000 ED patients with sepsis found that a one-hour delay in antibiotic therapy increased in-hospital mortality by 9%, with the highest mortality rates in patients treated within the first hour and in patients with septic shock [15]. A Dutch study carried out over 3.5 years also reported that guideline-adherent antibiotic therapy reduced in-hospital mortality by 5.8% in patients with severe sepsis and septic shock [16].

There are a few publications describing that hypoalbuminemia resulted in a significantly higher mortality rate and may serve as an indicator of severity and prognosis in sepsis [17,18]. Hypoalbuminemia may influence the pharmacokinetic parameters of the drugs, reducing pharmacodynamic effects, especially for time-dependent antibiotics [19].

To date, despite the incidence and importance of sepsis, no field studies have been conducted to assess the diagnosis, the first antibiotic treatment, and especially guideline adherence in patients admitted to the ED in Hungary.

The main purpose of this study was to evaluate the diagnostic accuracy and antibiotic prescription pattern for patients admitted to the ED due to sepsis, comparing Sepsis-2 to Sepsis-3 criteria. Additionally, we also aimed to identify risk factors that may influence clinical outcomes in sepsis.

## 2. Materials and Methods

### 2.1. Study Design

This single-center retrospective observational cohort study was conducted in the ED of an academic tertiary care medical center in Hungary. The study was approved by the local Research Ethics Committee Foundation (DE REB/IKEB: 6267-2022).

### 2.2. Inclusion and Exclusion Criteria

Patients admitted to the ED between January 2023 and December 2023 with clinically suspected sepsis or septic shock, and coded with sepsis (patients aged above 18 years), were identified through electronic records (UD MED System, hospital ward module, Debrecen, Hungary). Patients readmitted for recurrent sepsis within 30 days were excluded from the study. Further analysis encompassed the cases where sepsis diagnosis met Sepsis-2 or Sepsis-3 criteria.

### 2.3. Data Collection

Medical data were collected retrospectively on data collection forms designed by pharmacists and then recorded in Excel worksheets. Data collection included patients’ demographic characteristics such as age and gender, prior hospitalization, antibiotic use in the last month and at admission (agent selection, dosage, time of administration), present immunosuppressive drugs, comorbidities, clinical symptoms, and triage characteristics (category, time, and duration). Clinical and laboratory tests were collected as follows: arterial blood gas analysis, SIRS (Sever Immune Response Syndrome) parameters (body temperature, heart and respiratory rate, complete blood counts), SOFA (Sequential Organ Failure Assessment) score parameters (PaO_2_, FiO_2_, type of mechanical ventilation, platelet count, GCS (Glasgow Coma Scale), serum bilirubin and creatinine level, MAP-mean arterial pressure), serum lactate level, PCT (procalcitonin) level, WBC (white blood cells count), CRP (C-reactive protein) level, LDH (lactate dehydrogenase) level, IL-6 (interleukin-6) level, albumin level, systolic blood pressure, organ dysfunction, intravenous fluid or vasopressor administration. Data regarding admission and discharge time, triage (time, category, duration), clinical outcomes such as death (at the ED, in-hospital death, and 30-day mortality), and length of stay (at the ED: ED-LOS, and total: Total-LOS) were also recorded.

Since the exact time of the first dose of antibiotic administration was not revealed from the electronic documentation, we inferred it from the time spent by the patient at the ED (within 3 h, between 3 and 6 h, and between 6 and 24 h). Empirical antibiotic therapy was deemed adherent to guidelines when all antibiotics were appropriately selected based on the source of the infection (Table 1).

Patients were anonymized to ensure their identities remained confidential throughout the study.

### 2.4. Main Outcome Measures

Our primary outcome measure was to assess whether recorded sepsis diagnoses in the ED met the sepsis diagnostic criteria. In the present investigation, the accuracy of sepsis diagnosis was assessed by Sepsis-2 (International Sepsis Definitions Conference, 2001) and Sepsis-3 (The Third International Consensus Definitions for Sepsis and Septic Shock, 2016) criteria, respectively. Cases coded as sepsis but not meeting the aforementioned criteria were considered misdiagnosed. According to Sepsis-2 criteria, sepsis met the diagnostic criteria when SIRS ≥ 2 and a suspected or confirmed infection was present. Sepsis with organ dysfunction, hypotension, or hypoperfusion was also considered sepsis (severe sepsis), while sepsis with persistent hypotension despite adequate fluid resuscitation was defined as septic shock. As stated by Sepsis-3 criteria, sepsis met the diagnostic criteria when SOFA ≥ 2 (life-threatening organ dysfunction). The use of vasopressors required to maintain the MAP > 60 mmHg and lactate > 2 mmol/L after fluid resuscitation was defined as septic shock.

Secondly, we identified the risk factors and sources of infection associated with the incidence of sepsis in adult patients admitted to the ED, and their effect on clinical outcomes. CCI (Charlson Comorbidity Index) was used to assess the overall health condition of the patients, predicting 10-year survival in patients with multiple comorbidities. LOS (length of stay) indicated the duration of a single episode of hospitalization. The days of admission and discharge were each considered as a single day. ED-LOS reflected on the time (<24 h) spent at the ED.

Next, we assessed the triage flow (triage duration) and clinical outcomes (ED-LOS, LOS, 30-day mortality) of sepsis in different triage categories. Recorded triage categories (from red to white) were defined by the HETS (Hungarian Emergency Triage System), 2019.

Further, we also appraised the characteristics of empirical antibiotic administration (time, agent selection, guideline adherence) and its impact on clinical outcomes, being one of the secondary outcome variables. Guideline adherence was assessed on the basis of agent selection. The local guideline for sepsis is shown in Table 1.

Finally, we investigated the threshold values of different laboratory tests (PCT, WBC, CRP, LDH, and serum albumin and lactate levels) on admission to the ED and their impact on clinical outcomes in sepsis.

Data obtained by Sepsis-2 and Sepsis-3 criteria, in terms of main outcomes and measurements, were analyzed and compared.

### 2.5. Statistical Analyses

Descriptive statistics were used to describe the variables of the study. To compare categorical variables Fisher’s exact test was applied, while differences in continuous variables were assessed with a two-sample non-parametric *t*-test. Kaplan–Meier survival analysis was used to estimate how antibiotic therapy influences the survival/mortality rates. Statistical analyses were performed using GraphPad Software, LLC (version 8.4.3, 2020), *p*-values below 0.05 were considered statistically significant (* *p* ≤ 0.05; ** *p* < 0.001).

## 3. Results

**Figure 1:**
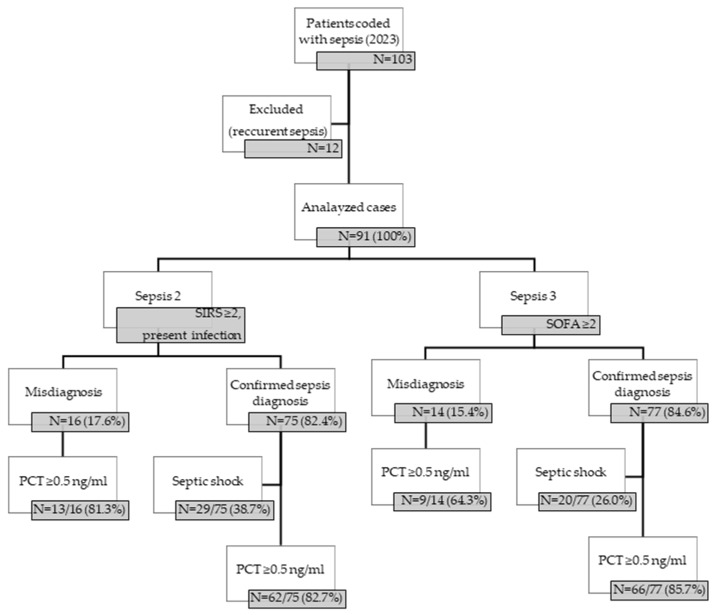
Algorithm used for sepsis diagnosis and diagnosis assessment. SIRS: Systemic Inflammatory Response Syndrome; SOFA: Sequential Organ Failure Assessment; PCT: procalcitonin.

The algorithm used for sepsis diagnosis and diagnostic assessment is shown in Figure 1. Based on both—Sepsis-2 and Sepsis-3—definitions, more than 80% (82.4% and 84.6%, respectively) of cases met the sepsis diagnosis, out of which more than 80% (82.7% and 85.7%, respectively) had PCT ≥ 0.5 ng/mL. There was no significant difference in PCT between the groups.

Further analyses were made only in cases with confirmed sepsis diagnosis.

### 3.1. Risk Factors and Sources of Infection

Patient characteristics and sources of infection are presented in Table 2.

In this study we have not found differences (Sepsis-2: 53.3% and Sepsis-3: 48.1% of males) in gender distribution between patient groups diagnosed with sepsis. However, the 30-day mortality rate was higher in males (67.5% and 67.6%, respectively).

In terms of age distribution, we observed that in both groups sepsis predominantly affected the elderly over 60 years of age (86.7% and 88.4%, respectively), and almost half of these were elderly aged 80+ (38.7% and 41.6%, respectively).

Regarding the overall health condition of the patients, we found that almost 80% (76.0% and 77.9%, respectively) of the patients had CCI > 4, which already predetermines an approximately 53% 10-year survival probability (Table 2). The most frequently observed comorbidities were diabetes mellitus and chronic liver/renal failure (between 33.3 and 39.0%), followed by dementia and connective tissue diseases (Supplementary Table S1).

Furthermore, hospitalization in the last month also increased the incidence of sepsis, affecting one-third (36.0% and 33.8%, respectively) of patients.

Appraising the sources of infection, we found that more than one third of sepsis cases presented with respiratory tract infection (RTI, Sepsis-2: 33.3% and Sepsis-3: 35.1%) or urinary tract infection (UTI, Sepsis-2: 37.3% and Sepsis-3: 33.8%), and more than 15% presented combined RTI and UTI (Sepsis-2: 17.3% and Sepsis-3: 16.9%). At the same time, the incidence of skin and soft tissue infection (SSTI) was below 10%. Occasionally, other infections (endocarditis and abdominal infections) have also occurred.

### 3.2. Risk Factors and Clinical Outcomes

Clinical outcomes in sepsis are presented in Table 3.

**Figure 2:**
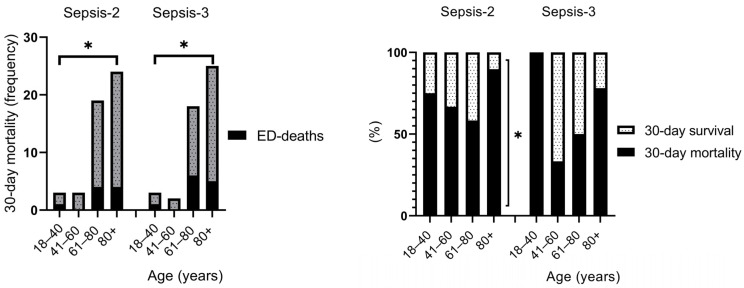
Mortality rate by age in sepsis (* *p* < 0.05).

We have not found significant differences between Sepsis-2 and Sepsis-3 in mortality rates. However, deaths in the ED and 30-day mortality rate was significantly higher (*p* < 0.05) in the age groups 60+ than in the younger age groups (Figure 2). Additionally, 30-day mortality rate was found to be higher than 30-day survival in sepsis, a result that was more sensistive for Sepsis-2 criteria (*p* < 0.05) (Figure 2). In both sepsis groups, the 90- and 180-day mortality is barely 12% higher than the 30-day mortality (77.3% vs. 65.3% and 74.0% vs. 62.3%, respectively) (Table 3).

We have not found significant differences between Sepsis-2 and Sepsis-3 in mortality rates. However, deaths in the ED and 30-day mortality rate was significantly higher (*p* < 0.05) in the age groups 60+ than in the younger age groups (Figure 2). Additionally, 30-day mortality rate was found to be higher than 30-day survival in sepsis, a result that was more sensistive for Sepsis-2 criteria (*p* < 0.05) (Figure 2).

**Figure 3:**
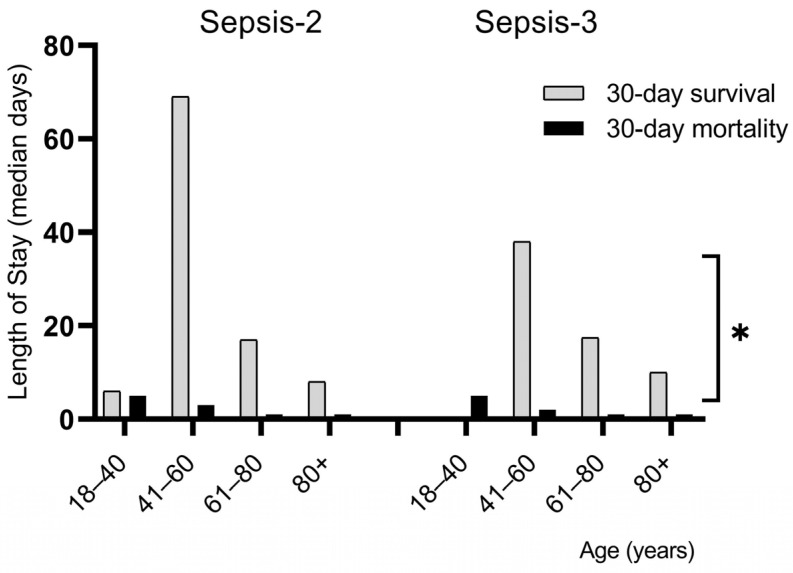
Length of stay by age in sepsis (* *p* < 0.05).

Although the assessment of LOS has not shown a significant difference in Sepsis-2 and Sepsis-3 (30-day mortality group: median 1 and 1 day, 30-day survival group: median 16 and 14 days, respectively), we observed that patients aged between 41 and 60 years spent the longest time in hospital, and the LOS decreased with age. As we expected, LOS was significantly higher in the 30-day survival group than in the 30-day mortality group (Table 2, Figure 3). CCI increased with age, but there were no significant differences in the 30-day mortality and 30-day survival groups, or in the Sepsis-2 and Sepsis-3 criteria (Table 3). 

### 3.3. Triage Flow (Triage Duration) and Clinical Outcomes

Triaging patients is a key part of emergency care. Triage duration and related clinical outcomes are presented in Table 4.

Based on both definitions of sepsis, the highest proportion (57.3% and 57.1%, respectively) of patients were placed in triage category 3. The median time to triage was 6–16 min and 6–13 min for Sepsis-2 and Sepsis-3, and the most severe category (I.) had the longest triage duration. According to Sepsis-2, patients in this category (I.) typically spent more time in the ED (median 7.4 vs. 4.2–5.2 h), and due to the deaths that occurred (100% ED-mortality), LOS was also typically lower in this category. Our observations were similar for Sepsis-3, except for patients from triage category I., who spent less time (4 vs. 7.4 h) on the ED than those with Sepsis-2. As expected, mortality rates decreased by increasing the number of triage categories. Nevertheless, LOS increased in higher categories (III.–IV.) with a decreasing tendency in category (IV.), suggesting that recovery time increases with the severity of sepsis (Table 4).

### 3.4. Empirical Antibiotic Administration and Clinical Outcomes

**Figure 4:**
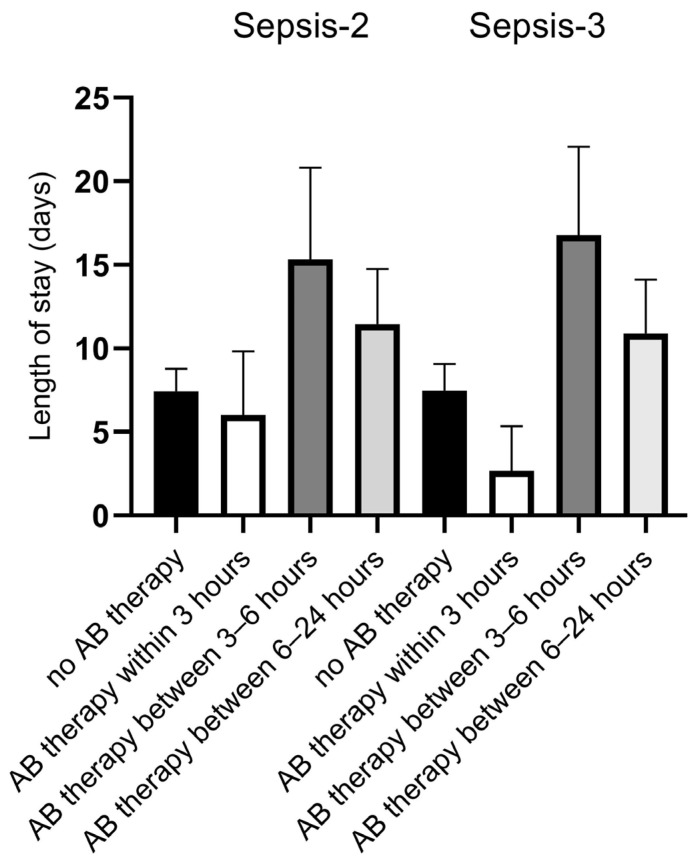
Length of stay and timing of antibiotic administration.

Our results show that for both Sepsis-2 and Sepsis-3, just over half of the patients (57.3% and 59.7%, respectively) received antibiotics in the ED, and the time of administration within 3 h was relatively low (9.3% and 6.5%, respectively) (Table 5). No significant difference was found in LOS and 30-, 90-, and 180-day mortality rates either between Sepsis groups or in terms of timing of AB administration (Table 5, Figure 4).

**Figure 5:**
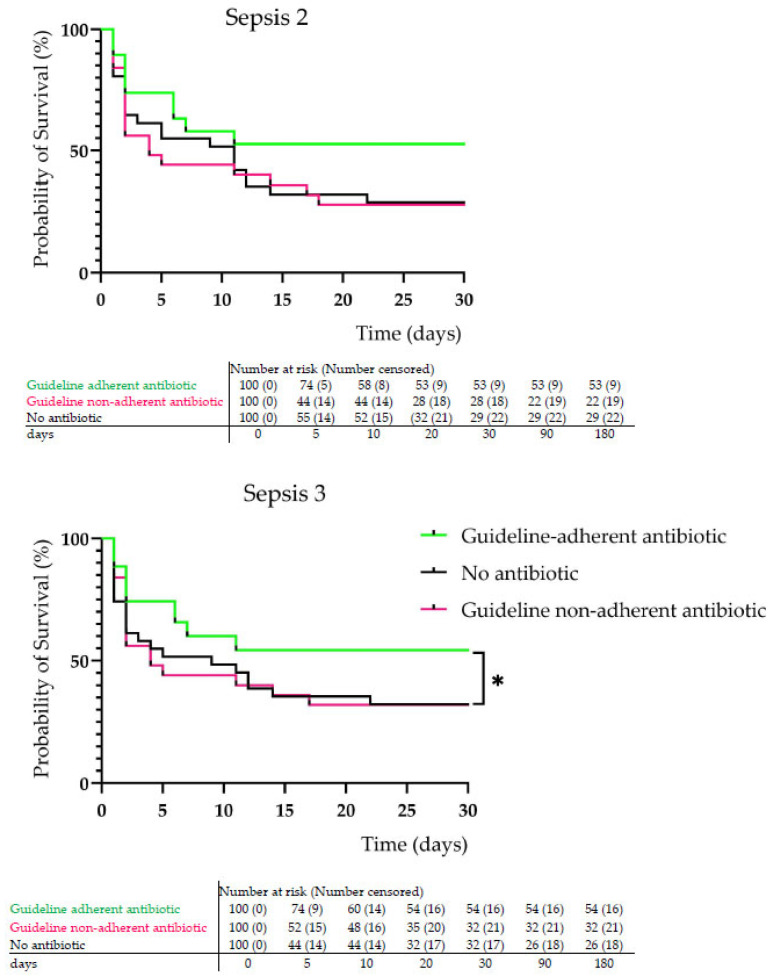
Survival analysis in sepsis regarding antibiotic therapy (* *p* < 0.05).

Assessing guideline adherence in the first empiric AB therapy of sepsis, we found that guideline non-adherent therapy occurred significantly more often than guideline-adherent therapy (for both sepsis groups 41% vs. 58%, respectively, *p* < 0.001), leading to higher 30-day mortality rate and shorter LOS (Table 6), but these differences were significant only in case of 90- and 180-day mortality. However, the survival analysis performed showed that in the Sepsis-3 group guideline adherence was associated with a significantly (*p* < 0.05) higher probability of survival than no AB or guideline non-adherent AB therapy (Table 6, Figure 5).

### 3.5. Investigated Laboratory Parameters in Sepsis

The laboratory parameters assessed were PCT, WBC, CRP, LDH, serum albumin, and lactate levels at admission in the ED. Although in each case in the 30-day mortality group the inflammatory markers and LDH were higher (and albumin lower) compared with the 30-day survival group, the differences were significant only in case of LDH (Sepsis-3: 325 vs. 227 mg/dL, respectively, *p* = 0.004) and albumin (Sepsis-2: 26 vs. 30 g/L, respectively, *p* = 0.050) levels (Table 7). A Benjamini–Hochberg correction applied on laboratory variables showed an FDR-corrected *p*  =  0.050.

## 4. Discussion

Sepsis remains a clinical condition with a potentially high mortality rate, and is often misdiagnosed, since currently there is no specific diagnostic biomarker or certain and specific method that can clearly identify it. Despite the fact that Sepsis-2 and Sepsis-3 definitions may help in prompt and early detection of sepsis, estimated missed or delayed diagnosis occurred between 8.2% and 20.8% [20]. At the same time, serum PCT levels increase proportionally with the severity of sepsis, but levels below 0.5 ng/mL may not exclude an infection [21].

Our single-center study results are in line with these findings, since based on Sepsis-2 and Sepsis-3 criteria, the rate of confirmed sepsis diagnosis was more than 80% (82.4% and 84.6%, respectively), and serum PCT levels were only higher in 80% (82.7% and 85.7%, respectively) of the cases.

### 4.1. Risk Factors and Clinical Outcomes

Several studies focus on risk factors that may influence clinical outcomes in sepsis. An American study carried out at the national level in 2011 found no difference in hospitalization rate for sepsis between genders. However, this rate increased with age, and patients aged over 85 years were 30 times more likely to be hospitalized for sepsis than those under 65 years [22]. According to a systematic review including 14 articles, older age, and male gender were associated with a higher risk of sepsis among ICU-admitted patients [23]. A retrospective cohort study in Japan using ED data found that older age and the presence of comorbidities were associated as potential risk factors in sepsis [24,25]. Similarly, a large prospective study in Atlanta found that age, comorbidities, and co-infections (pneumonia, malignancy, and UTI) were associated with early mortality in sepsis [24]. Furthermore, a retrospective cohort study identified an increased risk of sepsis in the case of prior (90-day) hospitalization with exposure to high-risk antibiotics [26].

In this study, we found no difference in gender, but the 30-day mortality rate was higher in males (67.5% and 67.6%, respectively, for Sepsis-2 and Sepsis-3). Similarly, in the literature, the elderly over 60 years of age were predominantly affected by sepsis (86.7% and 88.4%, respectively), half of these representing the elderly aged 80+ (38.7% and 41.6%, respectively). Additionally, both ED-deaths and 30-day mortality rates were significantly higher in these age groups (Sepsis-2 showing higher sensitivity in this analysis), leading to a shorter LOS. However, 30-day survivals in sepsis showed significantly higher LOS in all age groups for both Sepsis criteria. Regarding comorbidities in our investigation, almost 80% of the patients had CCI > 4, which is responsible for the high mortality rate. The majority of elderly patients were admitted to the ED in a serious clinical condition. The most common sources of infections were UTIs and RTIs. In addition, we also identified pre-hospitalization (in the last month) as a risk factor for sepsis.

Significant differences between Sepsis-2 and Sepsis-3 criteria were not found.

### 4.2. Triage Flow (Triage Duration) and Clinical Outcomes

Previous studies have shown that appropriate triage flow, with an early recognition of sepsis and early onset of therapy (antibiotic and fluid therapy), significantly contributed to improved clinical outcomes. Nevertheless, no differences were found in mortality rates [27,28]. In contrast, according to a Canadian study performed on patients admitted to the ED with sepsis after implementation of a centralized alert system (triage), survival increased significantly (*p* < 0.001), suggesting that a centralized triage flow has the potential to improve patient clinical outcomes in sepsis [29]. Another Californian study assessing the time of antibiotic administration found that due to the triage, the door-to-antibiotic time also decreased (by 33.4 min, from 105.3 to 71.9 min) without affecting triage time [30].

As for triage flow, our result shows a relatively low time frame for both sepsis criteria (median 6–16 and 6–13 min, respectively), and category I. had the longest triage duration with 100% mortality rate. Regarding the ED-LOS, Sepsis-2 showed higher sensitivity in this analysis, since patients in triage category I. spent a longer time in the ED than in Sepsis-3 (7.4 vs. 4 h, respectively), where no difference was found between triage categories. The relatively high mortality rate suggests ineffective risk estimation during the triage process.

Despite the implementation of the triage system in Hungary (2018), a relatively high number of patients presenting at the free-access ED (30%) do not require acute care, delaying triage. This can be further postponed by the lack of physicians and nurses, as well as the time required to calculate various sepsis scores [31]. Altogether, this may result in additional workload for ED staff, delaying the appropriate sepsis diagnosis.

### 4.3. Empirical Antibiotic Administration and Clinical Outcomes

Researchers agree that early AB administration plays a key role in the clinical outcome of sepsis. However, the optimal time of administration is controversial.

A prospective single-center cohort study found that 11.8% of sepsis cases had not received AB therapy in the ED, while guideline-adherent AB agent selection (started in median 3 (2:06–4:24) hours) counted for 68.1% and was associated with significantly shorter LOS (6 vs. 8 days) [32]. According to a retrospective cohort study performed on patients admitted with sepsis to the ED of a tertiary teaching hospital, the AB administration within three hours was significantly associated with higher in-hospital survival (0.54, 95% CI 0.34 to 0.87; *p* = 0.01), and shorter LOS (11 vs. 15 days) [33]. Furthermore, a prospective Danish observational cohort study results showed the lowest 28-day mortality in those ED patients with sepsis who received AB between 1 and 9 h, while AB administration within 1 h or beyond 9 h was accompanied by the highest mortality rate [34]. In a Korean prospective cohort, 77.8% of AB therapies administered in the ED were guideline-adherent; however, the time and guideline adherence were not associated with improved 7-, 14-, and 28-day mortality [35]. In addition, a Swedish retrospective cohort study found that only 47% of patients received full (agent selection and dosing) guideline-adherent antibiotic therapy. And while the time to first antibiotic administration was not associated with mortality, full guideline adherence resulted in reduced 30-day mortality [36].

In our study, the rate of AB therapy started in the ED is relatively low (57.3% vs. 59.7%, Sepsis-2 and Sepsis-3), and the reasons behind this are not clear. It is important to note that due to incomplete documentation of the first antibiotic administration time, patients assumed to receive antibiotics within 6 or 24 h may have received their first dose of antibiotic even within 3 h, limiting the accuracy of our assessment. Nevertheless, apart from that, in both Sepsis groups, the time of administration of the first empirical AB administration has no particular impact on clinical outcomes. While it would be unwise to draw conclusions regarding clinical outcomes in this case, the study made it clear that, in addition to sepsis diagnostics, it is definitely necessary to implement antibiotic time-stamping protocols, which may also contribute to improving sepsis outcomes.

At the same time, our investigation found a significantly lower rate of guideline-adherent AB agent selection (for both sepsis groups 41% vs. 58%, respectively, *p* < 0.001), leading to shorter LOS, which can be explained by a higher in-hospital mortality rate, decreasing the chance of recovery day by day. Survival analysis clearly showed that guideline adherence led to a significantly higher probability of survival (median 37 days) than guideline non-adherent AB administration (median 4 days). Interestingly, patients with no AB therapy in the ED had a higher survival probability (median 11 and 9 days, according to Sepsis-2 and Sepsis-3). Probably, the severity of sepsis was not as pronounced, since patients survived longer without AB than those with guideline non-adherent AB therapy. In addition, the probability of death was characteristic for the first 20 days, and day 10 represented the threshold time for survival in guideline adherence (Figure 5).

### 4.4. Investigated Laboratory Parameters in Sepsis

The challenges of diagnosing sepsis led to research for useful indicators with predictive values for sepsis clinical outcomes. Thus, some studies underscore the predictive value of elevated LDH levels and hypoalbuminemia in sepsis outcomes. A large Chinese study investigating the relationship between LDH levels and mortality in sepsis found that LDH is an independent risk factor for death in sepsis (1.005, 95% CI 1.002–1.007, *p* = 0.001) [37]. Furthermore, according to a longitudinal Indian study, the mortality rate was significantly elevated among sepsis patients with hypoalbuminemia compared to patients without (29.3% vs. 11.4%, *p* = 0.029) [17]. 

As for assessed laboratory parameters at admission in the ED, only LDH (Sepsis-3: median 325 vs. 227 mg/dL, *p* < 0.001) and serum albumin (Sepsis-2: median 26 vs. 30 g/L, *p* ≤ 0.05) levels show significant differences between 30-day mortality and 30-day survival groups, and seemed to be useful in the early diagnosis of sepsis regarding clinical outcomes. Although we have not found a significant relationship between lactate level and mortality in our study groups, there are publications which found it a useful indicator as well. Based on our research, LDH is a useful indicator to predict 30-day mortality in the ED, which may be attributed to the high rate of pneumonia as a source of infection in sepsis. Previous research demonstrated that an elevated LDH level signifies increased pneumonia-related mortality [38,39].

Regarding albumin, it plays a crucial role in terms of the pharmacokinetics and pharmacodynamics of drugs. Drugs bound to albumin, and only the free fraction will be distributed and excreted from the plasma as the central compartment. Therefore, hypoalbuminemia increases the apparent volume of distribution and clearance of the drug, which may reduce the achievement of pharmacodynamic goals, especially in the case of time-dependent antibiotics (beta-lactams indicated by the local guidelines) used in sepsis. It is assumed that maintaining the serum albumin level could have a positive effect on the clinical outcome of sepsis.

The combined use of biomarkers has been increasingly recognized for improving the sensitivity and specificity of sepsis detection, especially in the early stages. Benjamini–Hochberg analysis (FDR = 0.05) supported the association between elevated LDH levels, hypoalbuminemia, and the diagnosis of sepsis. Overall, these laboratory tests, when receiving attention as combined sepsis biomarkers, may be able to improve the early diagnosis and prognosis of sepsis.

### 4.5. Strengths and Limitations

Our single-center study results may not be generalized to other healthcare institutes. The retrospective data collection from the electronic medical system might contain inexactness and potential biases. However, most of the data needed for the study were recorded in detail.

The first limitation of the study was the relatively low sample size for sepsis in the study period. The method of data collection resulted in severe cases being more likely to be included in the sample than mild cases. However, this number of cases meets the minimum (N = 30) statistical requirement. Furthermore, in some cases (N = 3), not all SOFA parameters (bilirubin due to hemolysis, and PaO_2_) were retrievable from the electronic medical system. At the same time, in these cases, the SOFA score would not have reached the minimum score (2 points) for sepsis even with a positive test. Our second limitation was the lack of knowledge of the time of antibiotic administration, which has not been recorded by the physicians, and we just inferred it from the time spent in the ED. Moreover, assessing full guideline adherence was not possible because, in many cases, only the antibiotic agent selection was registered.

## 5. Conclusions

A substantial majority of patients recorded with sepsis in the Emergency Department met the Sepsis-2 and Sepsis-3 criteria. The most important identified risk factors in sepsis were older age (60+ years) and comorbidities (CCI ≥ 4). Elevated LDH (median 325 mg/dL) and decreased albumin levels (median 26 g/L) may be used as early indicators of sepsis severity. Although the time to first antibiotic administration was not associated with significantly better clinical outcomes, the guideline-adherent agent selection resulted in a significantly higher probability of survival. No significant differences were found between Sepsis-2 and Sepsis-3 in terms of diagnostic accuracy, survival, or mortality. Implementing a real-time checklist to identify patients at risk of developing sepsis, along with antibiotic timestamps, could decrease diagnostic and therapeutic delays.

## Figures and Tables

**Table 1:** Guideline-adherent empirical antibiotic therapy in sepsis at the ED.

Source of Infection	Guideline-Adherent Agent Selection
Respiratory tract infections (RTIs)	beta lactam + macrolide *ceftriaxone* + *clarithromycin/azithromycin*
Urinary tract infections (UTIs)	beta lactam + aminoglycoside *ceftriaxone* ± *amikacin*
Skin and soft tissue infection (SSTIs)	beta lactams
Endocarditis	beta lactam + beta lactamase inhibitor *amoxicillin + clavulanic acid*
Abdominal infections	beta lactam + nitroimidazole *ceftriaxone* ± *metronidazole*

**Table 2:** Patient characteristics and sources of infection.

Parameters	Confirmed Sepsis Diagnosis				*p*-Values
	Sepsis-2		Sepsis-3		
	N = 75	100%	N = 77	100%	
**Gender (Male)**	40	53.3	37	48.1	0.780
**Age**					
18–40 years	4	5.3	3	3.9	0.720
41–60 years	6	8.3	6	7.8	1.000
61–80 years	36	48.0	36	46.8	1.000
80+ years	29	38.7	32	41.6	0.880
**Antibiotic allergy**	9	12.0	8	10.4	0.804
**CCI-Charlson Comorbidity Index**					
0	2	2.7	1	1.3	0.620
1	2	2.7	2	0.3	1.000
2	4	5.3	4	5.2	1.000
3	2	2.7	3	3.9	1.000
4	8	10.7	7	9.1	0.794
>4	57	76.0	60	77.9	1.000
**Hospitalization in the last month**	27	36.0	26	33.8	0.874
**Source of infection**					
Respiratory tract infections (RTIs)	25	33.3	27	35.1	1.000
Urinary tract infections (UTIs)	28	37.3	26	33.8	0.874
Mixed (RTI and UTI)	13	17.3	13	16.9	1.000
Skin and soft tissue infection (SSTIs)	7	9.3	6	7.8	0.781
Endocarditis	1	1.3	1	1.3	1.000
Abdominal infections	1	1.3	2	2.6	1.000
Not known	0	0	2	2.6	0.497

**Table 3:** Clinical outcomes in sepsis.

Parameters	Sepsis-2		Sepsis-3		*p*-Values	Sepsis-2	Sepsis-3	*p*-Values
	N = 75	100%	N = 77	100%		CCI (Mean ± SD, Median)		
Discharged home	6	8	5	6.5	0.766	6.50 ± 2.07 (6)	6.80 ± 2.17 (6)	0.820
Moved to another hospital ward	49	65.3	49	63.6	1	6.49 ± 3.24 (6)	6.33 ± 3.02 (6)	0.797
Removed to ICU	11	14.6	11	14.3	1	5.46 ± 3.05 (7)	5.55 ± 3.05 (7)	1.000
**Death in the ED**	**9**	**12.0**	**12**	**15.6**	**0.646**	**7.44 ± 2.30 (7)**	**7.08 ± 2.33 (7)**	**0.721**
18–40 years	1/4	25.0	1/3	33.3	1	9.00 ± 0.00 (9)	9.00 ± 0.00 (9)	1.000
41–60 years	0/6	0.0	0/6	0.0	1	-	-	-
61–80 years	4/36	11.1	6/36	16.7	0.738	7.50 ± 3.51 (7.5)	6.67 ± 3.08 (5.5)	0.701
80+ years	4/29	13.8	5/32	15.6	1	7.00 ± 0.82 (7)	7.20 ± 0.84 (7)	0.729
**30-day mortality**	**49**	**65.3**	**48**	**62.3**	**0.897**	**6.67 ± 2.92 (7)**	**6.75 ± 2.88 (7)**	**0.897**
18–40 years	3/4	75.0	3/3	100.0	1	3.33 ± 4.93 (1)	3.33 ± 4.93 (1)	1.000
41–60 years	3/6	50.0	2/6	33.33	1	2.33 ± 1.16 (3)	2.00 ± 1.41 (2)	0.789
61–80 years	19/36	52.8	18/36	50.0	1	7.21 ± 2.70 (7)	7.17 ± 2.71 (7)	0.961
80+ years	24/29	82.8	25/32	78.1	1	7.21 ± 2.32 (7)	7.24 ± 2.28 (7)	0.962
**90- and 180-day mortality**	**58**	**77.3**	**57**	**74.0**	**0.902**	**6.81 ± 3.05 (7)**	**6.75 ± 2.92 (7)**	**0.932**
18–40 years	3/4	75.0	3/3	100	1	3.33 ± 4.93 (1)	3.33 ± 4.93 (1)	1.000
41–60 years	4/6	66.7	3/6	50.0	1	2.75 ± 1.26 (3)	2.00 ± 1.41 (2)	0.667
61–80 years	24/36	66.7	23/36	63.9	1	7.25 ± 2.86 (7)	6.91 ± 2.59 (7)	0.659
80+ years	27/29	93.1	28/32	87.5	1	7.41 ± 2.56 (7)	7.43 ± 2.52 (7)	0.909
**LOS 30-day mortality** **(mean ± SD, median-days)**	4.31 ± 5.39 (1)		3.60 ± 5.03 (1)		0.509	6.67 ± 2.92 (7)	6.75 ± 2.88 (7)	0.897
**LOS 30-day survival group** **(mean ± SD, median-days)**	22.88 ± 20.36 (16)		22.93 ± 21.61 (14)		0.994	6.08 ± 3.25 (6)	5.72 ± 2.75 (5)	0.665
18–40 years	6.00 ± 0.00 (6)		-		-	-	-	-
41–60 years	52.00 ± 41.22 (69)		40.75 ± 40.48 (38)		0.732	5.33 ± 4.16 (4)	4.50 ± 3.79 (3)	0.793
61–80 years	22.53 ± 14.23 (17)		23.67 ± 18.4 (17.5)		0.840	6.06 ± 2.79 (6)	5.56 ± 2.31 (5.5)	0.564
80+ years	10.00 ± 4.06 (8)		10.86 ± 4.26 (10)		0.734	7.80 ± 3.56 (7)	6.86 ± 3.24 (6)	0.643

CCI: Charlson Comorbidity Index; ICU: Intensive care unit; ED: Emergency Department; LOS: length of stay/total hospitalization; SD: standard deviation.

**Table 4:** Triage flow (triage duration) and clinical outcomes.

Parameters Sepsis 2 (N = 75) Sepsis 3 (N = 77)	Triage Category				
	I. N = 3 (4.0%) N = 4 (5.2%)	II. N = 21 (28.0%) N = 23 (29.9%)	III. N = 43 (57.3%) N = 44 (57.1%)	IV. N = 8 (10.7%) N = 6 (7.8%)	*p*-Values 1.000
**Triage duration** (mean ± SD, median in minutes) Sepsis 2 Sepsis 3	19.67 ± 9.74 (16) 15.5 ± 11.10 (13)	11.05 ± 14.23 (6) 10.43 ± 13.83 (6)	13.47 ± 23.83 (8) 13.0 ± 23.64 (8)	11.50 ± 5.32 (13.5) 9.33 ± 4.46 (9)	0.522
**ED-LOS** (mean ± SD, median in hours) Sepsis 2 Sepsis 3	7.25 ± 5.30 (7.4) 5.25 ± 5.21 (4)	4.80 ± 3.38 (4.2) 4.65 ± 3.04 (5)	6.34 ± 3.56 (5.2) 5.80 ± 3.53 (5)	7.03 ± 6.48 (4.5) 4.0 ± 1.63 (4.5)	0.393
**ED-mortality**					
Sepsis 2	3/3 (100%)	2/21 (9.5%)	4/43 (9.3%)	0/8 (0%)	0.955
Sepsis 3	4/4 (100%)	4/23 (17.4%)	4/44 (9.1%)	0/6 (0%)	
**Total LOS** (mean ± SD, median in days) Sepsis 2 Sepsis 3	0.33 ± 0.47 (0) 0.25 ± 0.43 (0)	6.33 ± 11.37 (1) 8.61 ± 16.42 (1)	13.93 ± 17.76 (10) 13.32 ± 17.74 (9)	9.13 ± 4.20 (8.5) 8.83 ± 4.22 (7.5)	0.888
**30-day mortality** Sepsis 2 Sepsis 3	3/3 (100%) 4/4 (100%)	16/21 (76.2%) 16/23 (69.6%)	31/43 (72.1%) 27/44 (61.4%)	4/8 (50.0%) 2/6 (33.3%)	0.891
**90- and 180-day** **mortality** Sepsis 2 Sepsis 3	3/3 (100%) 4/4 (100%)	18/21 (85.7%) 18/23 (82.6%)	32/43 (74.4%) 32/44 (72.7%)	5/8 (62.5%) 2/6 (33.3%)	0.761

SD: standard deviation; ED-LOS: length of stay at the Emergency Department; ED-mortality: mortality at the emergency department; LOS: length of stay/total hospitalization.

**Table 5:** Characteristics of antibiotic therapy in sepsis.

Parameters	Frequency Sepsis 2: N = 75, 100% Sepsis 3: N = 77, 100%	30-Day Mortality Sepsis 2 Sepsis 3	*p*-Values	90- and 180-Day Mortality Sepsis 2 Sepsis 3	*p*-Values	LOS (Mean ± SD, Median-Days) Sepsis 2 Sepsis 3	*p*-Values
**Antibiotic therapy**							
no antibiotic therapy at the ED	32 (42.7%) 31 (40.3%)	22/32 (68.8%) 21/31 (67.7%)	0.678	25/32 (78.1%) 24/31 (77.4%)	0.434	7.44 ± 7.52 (6.5) 7.48 ± 8.54 (4)	0.860
antibiotic therapy at the ED	43 (57.3%) 46 (59.7%)	27/43 (62.8%) 27/46 (58.7%)		33/43 (76.7%) 33/46 (71.7%)		13.21 ± 18.83 (5) 13.17 ± 19.82 (5)	
**Administration time**			Sepsis-2: N = 43 (100%) Sepsis-3: N = 46 (100%)				
within 3 h	4/43 (9.3%) 3/46 (6.5%)	2/4 (50.0%) 2/3 (66.6%)	0.800	3/4 (75.0%) 3/3 (100%)	0.342	6.00 ± 7.66 (4) 2.67 ± 4.62 (0)	0.382
between 3 and 6 h	20/43 (46.5%) 22/46 (47.8%)	13/20 (65.0%) 12/22 (54.5%)		17/20 (85.0%) 16/22 (72.7%)		16.00 ± 23.55 (4.5) 16.77 ± 24.88 (6)	
between 6 and 24 h	19/43 (44.2%) 21/46 (45.7%)	12/19 (63.2%) 13/21 (61.9%)		13/19 (68.42%) 14/19 (73.7%)		11.79 ± 15.15 (6) 10.90 ± 14.67 (5)	

LOS: length of stay-total hospitalization; SD: standard deviation; ED: Emergency Department.

**Table 6:** Clinical outcomes and probability of survival in sepsis.

Parameters	No AB Therapy	Guideline Adherent AB Therapy	Guideline Non-Adherent AB Therapy	*p*-Values
**Frequency**				
Sepsis 2	32/75 (42.7%)	18/43 (41.9%)	25/43 (58.1%)	<0.001 **
Sepsis 3	31/77 (40.6%	19/46 (41.3%)	27/46 (58.7%)	
**Death at the ED**				
Sepsis 2	5/32 (15.6%)	2/18 (11.1%)	2/25 (8.0%)	0.136
Sepsis 3	7/31 (22.6%)	3/19 (15.8%)	2/27 (7.4%)	
**30-day mortality**				
Sepsis 2	22/31 (71.0%)	9/19 (47.4%)	18/26 (69.2%)	0.058
Sepsis 3	21/31 (67.7%)	10/19 (52.6%)	17/27 (63.0%)	
**90- and 180-day mortality**				
Sepsis 2	25/31 (80.6%)	11/19 (57.9%)	22/26 (84.6%)	0.041 *
Sepsis 3	24/31 (77.4%)	12/19 (63.2%)	21/27 (77.8%)	
**LOS** **(mean ± SD, median-days)**				
			
Sepsis 2	7.43 ± 7.54 (6.5)	13.72 ± 18.03 (7)	12.84 ± 19.71 (3)	0.051
Sepsis 3	7.48 ± 8.85 (4)	11.26 ± 16.98 (6)	14.52 ± 21.49 (4)	
**Probability of survival**				
**Log-rank (Mantel–Cox) test**	X^2^ = 3.342			
**(median survival—days)**	X^2^ = 4.131			
Sepsis 2	11 vs. 37 vs. 4			0.181
Sepsis 3	9 vs. 37 vs. 4			0.042 *

AB: antibiotic; ED: Emergency Department; LOS: length of stay/total hospitalization; * *p* ≤ 0.05; ** *p* < 0.001.

**Table 7:** Laboratory parameters and their threshold values in sepsis.

Parameters (Mean ± SD, Median)	Confirmed Sepsis Diagnosis Sepsis 2 (N = 75) Sepsis 3 (N = 77)		
	30-Day Mortality N = 49/75 N = 48/77	30-Day Survival N = 26/75 N = 29/77	*p*-Values
PCT (ng/mL)	27.80 ± 83.35 (3.8) 33.33 ± 87.49 (4.9)	76.95 ± 228.1 (3.8) 110.2 ± 255.9 (2.9)	0.191 0.067
WBC (G/L)	18.98 ± 16.73 (17.1) 19.57 ± 17.51 (17.4)	19.63 ± 18.41 (16.8) 18.19 ± 16.91 (15.2)	0.989 0.735
CRP (mg/dL)	217.2 ± 129.5 (195.3) 231.2 ± 131.7 (205.6)	169.0 ± 124.1 (141.3) 205.0 ± 134.5 (179.4)	0.149 0.405
LDH (mg/dL)	393.00 ± 326.5 (267) 458.1 ± 367.0 (325)	252.5 ± 82.50 (255) 236.6 ± 84.99 (227)	0.071 0.004 *
Albumin (g/L)	26.12 ± 6.62 (26) 26.21 ± 6.06 (26)	29.81 ± 6.06 (30) 28.55 ± 7.41 (28)	0.050 * 0.191
lactate (mmol/L)	5.11 ± 4.02 (3.5) 5.17 ± 3.94 (4)	3.59 ± 2.99 (2.4) 3.34 ± 2.92 (2.4)	0.170 0.067

SD: standard deviation; PCT: procalcitonin; WBC: white blood cells; CRP: C-reactive protein; LDH: lactate dehydrogenase enzyme; * *p* ≤ 0.05.

## Data Availability

Data are available from the corresponding author upon reasonable request.

## Supplementary Materials

The following supporting information can be downloaded at: https://www.mdpi.com/article/10.3390/biomedicines13071566/s1, Table S1: Comorbidities in sepsis.

## Abbreviations

The following abbreviations are used in this manuscript:

CCI	Charlson Comorbidity Index
ECDC	European Center for Disease Prevention and Control
ED	Emergency Department
HAI	Hospital-acquired Infection
HETS	Hungarian Emergency Triage System
ICU	Intensive Care Unit
LOS	Length of Stay
qSOFA	Quick Sequential Organ Failure Assessment
RTI	Respiratory Tract Infection
Sepsis-2	International Sepsis Definitions Conference, 2001
Sepsis-3	Third International Consensus Conference 2016
SIRS	Systemic Inflammatory Response Syndrome
SOFA	Sequential Organ Failure Assessment
SSTI	Skin and Soft Tissue Infection
UTI	Urinary Tract Infection
