# Supplementary material for: Evaluation of the Diagnosis and Antibiotic Therapy of Sepsis in the Emergency Department: A Retrospective Observational Study
Source: Biomedicines. 2025 Jun 26;13(7):1566. doi: 10.3390/biomedicines13071566 (PMC12293071; doi:10.3390/biomedicines13071566)
Supplement: Supplementary file 1 [file biomedicines-13-01566-s001.zip › biomedicines-3642389-supplementary.pdf]

biomedicines

Table S1. Comorbidities in sepsis.

Comorbidities	Confirmed sepsis diagnosis				*p*-value
	Sepsis-2		Sepsis-3		
	N=75	100%	N=77	100%	
**Cardiovascular disease**	12	16.0	13	16.9	1
Diabetes mellitus	29	38.7	27	35.1	0.876
Chronic pulmonary disease	9	12.0	8	10.4	0.804
Chronic liver/kidney disease (moderate to severe)	25	33.3	30	39.0	0.640
Solid tumor	15	20.0	13	16.9	0.837
Localized	5	6.7	4	5.2	0.746
Metastatic	10	13.3	9	11.7	0.813
Peripheral vascular disease	15	20.0	13	16.9	0.837
Dementia	23	30.7	23	29.9	1
Peptic ulcer disease	1	1.3	1	1.3	1
Cerebrovascular accident and transient ischemic attack	19	25.3	19	24.7	1
Connective tissue disease	25	33.3	22	28.6	0.739
